# Exercise and the Cisd2 Prolongevity Gene: Two Promising Strategies to Delay the Aging of Skeletal Muscle

**DOI:** 10.3390/ijms21239059

**Published:** 2020-11-28

**Authors:** Yuan-Chi Teng, Jing-Ya Wang, Ya-Hui Chi, Ting-Fen Tsai

**Affiliations:** 1 Department of Life Sciences and Institute of Genome Sciences, National Yang-Ming University, Taipei 11221, Taiwan; andrea_chi@hotmail.com; 2 Institute of Biotechnology and Pharmaceutical Research, National Health Research Institutes, Zhunan 35053, Taiwan; jyw@nhri.edu.tw; 3 Institute of Molecular and Genomic Medicine, National Health Research Institutes, Zhunan 35053, Taiwan; 4 Aging and Health Research Center, National Yang-Ming University, Taipei 11221, Taiwan

**Keywords:** aging, skeletal muscle, exercise, metabolism, Cisd2

## Abstract

Aging is an evolutionally conserved process that limits life activity. Cellular aging is the result of accumulated genetic damage, epigenetic damage and molecular exhaustion, as well as altered inter-cellular communication; these lead to impaired organ function and increased vulnerability to death. Skeletal muscle constitutes ~40% of the human body’s mass. In addition to maintaining skeletal structure and allowing locomotion, which enables essential daily activities to be completed, skeletal muscle also plays major roles in thermogenesis, metabolism and the functioning of the endocrine system. Unlike many other organs that have a defined size once adulthood is reached, skeletal muscle is able to alter its structural and functional properties in response to changes in environmental conditions. Muscle mass usually remains stable during early life; however, it begins to decline at a rate of ~1% year in men and ~0.5% in women after the age of 50 years. On the other hand, different exercise training regimens are able to restore muscle homeostasis at the molecular, cellular and organismal levels, thereby improving systemic health. Here we give an overview of the molecular factors that contribute to lifespan and healthspan, and discuss the effects of the longevity gene Cisd2 and middle-to-old age exercise on muscle metabolism and changes in the muscle transcriptome in mice during very old age.

## 1. Introduction

Aging is inevitable and affects all living organisms. According to the first World Report on Aging and Health published in 2015, “At a biological level, ageing is associated with the gradual accumulation of a wide variety of molecular and cellular damage. Over time, this damage leads to a gradual decrease in physiological reserves, an increased risk of many diseases, and a general decline in the capacity of the individual. Ultimately, it will result in death.” Due to the complexity of these biological changes that lead to the functional decline of various organs, the biological changes associated with aging are “neither linear nor consistent, and they are only loosely associated with age in years”. In addition to functional deterioration, aging is the primary risk factor for a range of human pathogenic diseases, including the development of cancer, diabetes, cardiovascular disorders and various neurodegenerative diseases [1].

The disconnection between age and aging is prominently evidenced in patients who suffer from premature aging diseases. These heritable mutations that accelerate the onset of multiple aging phenotypes are termed segmental progeroid syndromes, because they accelerate aspects of natural aging [2]. For example, Hutchinson-Gilford progeria syndrome (HGPS) is caused by a splicing error that affects the LMNA gene; this results in organ-specific aging-like symptoms such as alopecia, atherosclerosis, increased joint immobility, osteolysis, severe lipodystrophy, scleroderma and various degrees of skin hyperpigmentation [3,4,5,6]. Such HGPS affected individuals have a mean lifespan of 13 years [3], and the patient cells show the characteristic molecular hallmarks of aging, including a delayed DNA-damage response, reduced telomere length, reduced mitochondrial function and altered chromatin organization. Several other premature aging diseases observed in humans and mice with defects in genome maintenance suggest that one of primary causes of natural aging is driven by genome instability attributable to oxidative stress [2].

At the same time, the primary hallmarks of aging, including genomic instability, telomere attrition, epigenetic alternations and loss of proteostasis, unequivocally promote organismal aging, various antagonistic aging hallmarks, including deregulated nutrient sensing, mitochondrial dysfunction and cellular senescence, have opposing effects depending on their intensity [1]. For example, optimal nutrient sensing and anabolism are important for survival. However, chronic excess uptake of nutrients or various metabolic dysfunctions is pathological [1]. Another example is cell senescence, which brings about permanent cell cycle arrest and has been described as a mechanism to limit cancer cell growth; however, this also increases the production of inflammatory cytokines. The latter can exhaust the pool of immune cells in the body [7]. These aging hallmarks occur together during aging and are interconnected; therefore, understanding their exact causal network is important [1].

## 2. Exercise and Aging

Accumulated evidence suggests that healthspan can be promoted by mimicking caloric/dietary restriction, tuning cellular and organismal stress responses, and affecting the metabolism of the microbiome, which in turn impacts on the host and/or modulates repair pathways, among other changes [8]. Of note among these, lifetime or regular exercise training is able to maintain many of the properties of muscle that are negatively affected by aging [9]. Sarcopenia, an age-related decline in muscle mass and function, is considered to be the main risk factor for the elderly and it directly related to disability, weakness, loss of independence and various health-related negative outcomes, including morbidity and mortality [10,11,12]. Thus, it seems that, by accumulating muscle mass during middle age, an individual may be able to prevent the functional loss that affects their muscles in later life [13]. Here we discuss the correlation between muscle aging and interventions that potentially might delay this process.

### 2.1. Exercise and Metabolism

Sufficient energy is required to support normal metabolic functions, growth, and tissue repair as well as physical activity. It is mainly provided by the oxidation of dietary and stored carbohydrates (CHOs) and fat. Theoretically, total daily energy expenditure consists of several different components: expenditure at the basal level or the resting stage, physical activity, and maintenance of the core body temperature by thermoregulation and the thermic effect of food uptake [14]. Energy expenditure is measured by indirect calorimetry calculated from the amount of O_2_ consumed (i.e., VO_2_) and the amount of CO_2_ produced (i.e., VCO_2_). The measurement of VO_2_ and VCO_2_ can be performed with great accuracy; during this process, the substrate (CHO or fat) oxidized as energy sources can be distinguished from each other using the ratio of O_2_ consumption to CO_2_ production (called the respiratory exchange ratio: RER) [14]. An RER of 1.0 is indicative of carbohydrates being the predominant fuel source, while a value of 0.7 indicates that fat is the predominant fuel source. Finally a value between 0.7 and 1.0 suggests a mix of both fat and carbohydrates [15].

#### 2.1.1. Resting Metabolism

Resting energy expenditure (REE) is the amount of energy expended by an organism at rest. REE accounts for between 60% and 70% of total energy expenditure (TEE). Historically, obese individuals were believed to have lower energy expenditure (EE) rates than non-obese individuals (normal and overweight). It was observed that REE is related most directly to the amount of lean mass, because lean mass is more metabolically active than fat tissue. Nevertheless, current evidence has revealed that obesity is unlikely to be a result of a consistently lower daily EE or REE [16], because obese persons tend to have increased amounts of fat-free mass to support their increased fat mass. Recent studies have indicated that REE, adjusted for fat-free mass, which mainly contains skeletal muscles, is normal in both obese persons as well as normal-weight persons who are predisposed to obesity [17,18]. On the other hand, sustained weight loss may not always result in a substantial and disproportionately low REE [19]. Even among those of similar weight, age and gender, REE varies markedly. However, men generally have higher REE values than women, even after adjusting for body weight and fat-free mass. In addition to the gender effect, increasing age is associated with a decreasing REE; this is due to at least in part age-dependent loss of fat-free mass [20].

#### 2.1.2. Exercise Metabolism

Exercise alters a variety of cell types and is involved in multiple signaling pathways that regulate systemic metabolism. During exercise, the relative levels of oxidation of fat and carbohydrate may be influenced by a range of factors, such as diet, muscle glycogen content, exercise intensity/duration, type of exercise and fitness status [21]. An acute bout of exercise increases the glucose uptake of skeletal muscle, while chronic exercise training improves mitochondrial function, promotes mitochondrial biogenesis and upregulates the expression of glucose transporter proteins and numerous other metabolic genes [22].

During low-intensity, steady-state exercise, RER values are typically between 0.80 and 0.88, meaning that fatty acids are the primary fuel. As the intensity of the exercise increases, carbohydrates become the dominant or primary fuel and the RER increases to between 0.9 and 1.0. However, strenuous exercise gives rise to hyperventilation and increased a blood lactate level, which is released from skeletal muscles, resulting in an elevation of CO_2_ production. Under this circumstance, the RER would exceed 1.0 and no longer reflect systemic substrate utilization [23]. RER reproducibly increases during exercise and therefore it has been identified as a parameter that can document maximal effort. The rate at which oxygen consumption adjusts to and recovers from a bout of exercise is associated with certain metabolic processes, including muscle storage of phosphocreatine and ATP, the replenishing of myoglobin and hemoglobin as well as the removal of metabolic waste. The ability of an individual to utilize these processes determines the speed at which oxygen consumption rises and falls, and thus these have been found to be associated with the health condition of a given individual [24,25].

#### 2.1.3. Impact of Middle-to-Old Age Exercise on Metabolism

**Figure 1:**
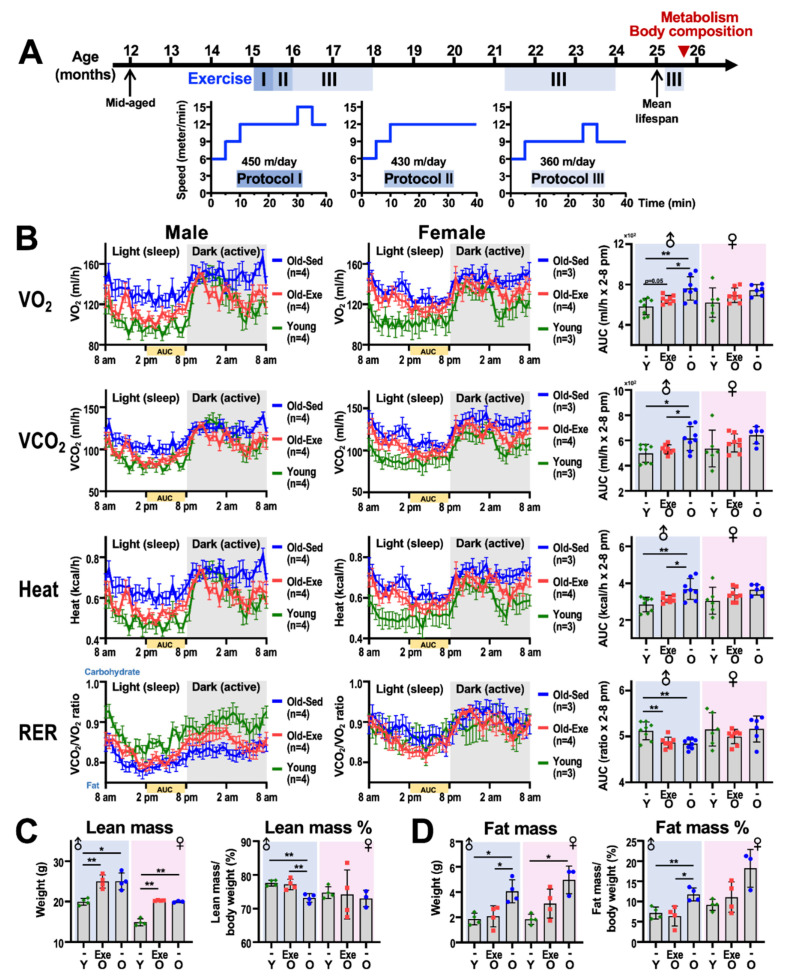
Systemic metabolism and body composition in mice during long-term exercise. (**A**) A schematic representation of the forced treadmill exercise protocol that was applied to mice when they reached 15-months of age. A ten-lane motorized rodent treadmill (SINGA Technology Corporation, Taipei, Taiwan) with 0^o^ of inclination was utilized for the exercise training. The exercise group of animals was trained on the treadmill; they ran 5 days/week for two 12-weeks cycles. In the first two weeks, the mice ran 450 m in 40 min per day. In the third and fourth week, the mice ran 430 m per day. Finally, from the fifth week, the mice ran 360 m per day. The running protocol was adjusted according to the performance of the mice on the treadmill. The sedentary mice were brought to the exercise location in order to expose these mice to the same conditions as the exercised mice. The Old-Exe group was rested for 3 months before initiating the second cycle of 12-weeks exercise. The animal experimental procedures were reviewed and approved by Institutional Animal Care and Use Committees (IACUC) of NHRI. (**B**) Whole-body metabolism of 25–26 months old male and female mice (exercise and sedentary) are shown in (**A**). The metabolism parameters were acquired using a metabolic cage system (TSE systems, Inc. Chesterfield, MO, USA). Mice were adapted to the cage for more than 48 h before measurement began. The metabolism parameters were recorded over consecutive 48-h periods, and the values during the light and dark hours were averaged from the 2-days of measurements. The mouse behavior and metabolism experiments were performed by specialists in the Taiwan Mouse Clinic, Academia Sinica. All the experiments were executed by following the standard operating procedures approved by the Taiwan Mouse Clinic (http://tmc.sinica.edu.tw/sops.html). The left and middle panels show a 24-h rhythm of oxygen consumption (VO_2_), carbon dioxide production (VCO_2_), heat production, and respiratory exchange ratio (RER) for the male and female mice, respectively. The data are presented as means±SEM. The right panel shows the area under curve (AUC) quantification of VO_2_, VCO_2_, heat and RER from 2 pm to 8 pm, a period during which mouse physical activity is low, which implies that this represents the metabolic rate at resting. AUC is presented as means ± SD. (**C**,**D**) Lean mass (**C**) and fat mass (**D**) of the exercised and sedentary old mice, compared to 3-month-old young mice without exercise. Net mass is presented in the left panel, and percent mass is presented in the right panel. The lean and fat mass values were measured using a Bruker Minispec LF50 TD (time-domain)-NMR Body Composition Analyzer (Bruker, Billerica, MA, USA). The data are presented as means ± SD. Statistical analysis used the Student’s *t*-test. *, *p* < 0.05; **, *p* < 0.01. Exe, exercise; Sed, sedentary; Y, young; O, old.

We sought to determine the impact of middle-to-old age regular exercise on energy metabolism in very old mice (>24 months, which is equivalent to >80 years old in human) [26]. We adopted a forced treadmill exercise protocol that has been shown to produce an overt effect on the cardiorespiratory and metabolic benefits similar to those observed in humans while exercising [27,28]. Male and female C57BL/6 mice at 15-month-old of age (equivalent to a 50-year-old in humans) were subjected to two cycles of 12-weeks forced treadmill exercise on a 5-on-2-off schedule. Mice were rested for three months before the initiation of a similar second cycle of 12-weeks exercise. The exercise intensity was reduced after the third week, and was further modified according to the physical performance of the mice on treadmill (Figure 1A). The energy metabolism of the exercise mice and the sedentary mice were monitored using a TSE metabolic cage system, and the metabolic parameters of 3-months old (i.e., young) mice that had never been on a treadmill were also measured as a comparison. Exercise did not seem to result in a significant impact on the oxygen consumption and CO_2_ production of the female mice. On the other hand, the effect of the animal’s circadian rhythm on oxygen consumption and heat production, when the dark and light hours of male mice are compared, appears to have a more prominent effect on the exercise group than the sedentary group (Figure 1B). In addition, the VO_2_ of exercised mice (i.e., Old-Exe) is closer to the young group than the sedentary mice (Old-Sed) when male mice were considered. On the other hand, the RER values of the male Old-Exe group are higher than the male Old-Sed group during the active hours; however this observation was not seen for the female mice (Figure 1B). These findings suggest that regular exercise is able to prompt the male mouse body to use carbohydrates as a fuel source during the active hours, but this phenomenon does not seem to occur in female mice.

### 2.2. Exercise and Body Composition

#### 2.2.1. Body Composition Measurement Techniques

At the molecular level, the human body is mainly composed of four components: water, fat, proteins and minerals [29]. A number of different techniques, both invasive and non-invasive, have been developed to allow body composition to be assessed. These techniques range from simple indirect measures, such as the waist-to-hip ratio, to more accurate direct volumetric measurements based on three-dimensional imaging [30]. The most commonly used methods for body composition measurement are bioelectrical impedance analysis, dilution techniques, air displacement plethysmography, dual energy X-ray absorptiometry (DEXA) and magnetic resonance imaging (MRI). These techniques allow us to measure fat (white and brown adipose tissues), lean mass, bone mineral content, total body water, extracellular water and various adipose tissue sub-depots (namely visceral, subcutaneous and intermuscular), skeletal muscle, selected organs and ectopic fat depots [31,32]. The measurements from DEXA and quantitative MRIs showed good agreement; they have linear correlations ranging from 0.97 and 0.99, and coefficients of variation ranging from 4.5% and 4.6% for fat (computed from adipose tissue, AT) and lean tissue (LT), based on a UK Biobank imaging cohort study of 4753 subjects. Quantitative MRIs also are able to measure muscle volumes and adipose tissues that ectopically infiltrated into skeletal muscles. When combined with rapid scanning protocols and efficient image analysis, quantitative MRI becomes a powerful tool for advanced body composition assessment [30].

#### 2.2.2. Body Composition and Aging

Human body composition plays an important role in quantifying health and nutritional status, assessing the impact of disease, and measuring changes due to nutritional, therapeutic or behavioral interventions. Body composition during aging is characterized by an increase in fat mass and decrease in lean tissues, including skeletal muscle mass, which in older adults results in reduced muscle strength and poorer functional capabilities, as well as greater morbidity and mortality [32].

Using a Bruker Minispec LF50 TD (time-domain)-NMR Body Composition Analyzer, we assessed the impact of exercise at middle-to-old age on the body composition of very old (25–26 months) mice. Three components of the mouse body were obtained; these were fat, free body fluid and lean tissue. Compare to the Young group, the reduction in percent lean mass was significant for the Old-Sed male mice (*p* < 0.01, Figure 1C), and this was paralleled by a significant increase in fat mass (*p* < 0.05, Figure 1D). Exercise during middle-to-old age among male mice was able to maintain the percentage of lean mass well, and the percentage of fat mass remained similar to that of the young group. Intriguingly, net lean mass and net fat mass both increased among the Old-Sed male mice. Among female mice, middle-to-old age slightly reduced the percentage of fat mass (*p* < 0.08) compared to their sedentary cohorts (Figure 1D), whereas the percent lean mass did not differ much between the young, Old-Exe and Old-Sed groups (Figure 1C). These findings indicate that exercise during middle-to-old age has different physiological consequences on the body composition of male and female mice.

## 3. Exercise and Sexual Dimorphism

Sexual dimorphism during exercise has been demonstrated and results in higher glycerol and free fatty acid (FFA) responses among women compared to men, together with a higher carbohydrate oxidation rate among men. Women seem to have a greater reliance on lipid metabolism during exercise, while men exhibit a preference for carbohydrate utilization [33]. The mechanisms that are able to increase the lipolytic rates of women include a higher total fat mass, an enhanced lipolytic sensitivity to epinephrine and an increased activation of the β-adrenergic receptors [34].

In a study that evaluated the impact on C57BL/6 mice of sexual dimorphism and of the estrous cycle on muscle strength and running power using Ergospirometry (cardiopulmonary exercise testing) [35], the results indicated that thermoregulation was more efficient in male mice than in female mice. On the other hand, exercise-induced increase of O_2_ consumption and CO_2_ production were similar between the sexes. In females, pro-estrus impaired economy during running and estrus impaired exercise heat loss [36]. This study also indicated that these sex-associated phenomena that respond to exercise disappeared after further normalization of exercise performance by body weight. Previous studies have revealed that body weight, lean mass and muscle strength are secondary sexual characteristics and therefore justification factors need to be considered when comparing the differences in the running performance between the sexes [36].

Gender is a key determinant for resting lipid metabolism. Additionally, there is heterogeneity of post-exercise resting metabolism in humans. A meta-analysis has indicated that men experience a greater increase in resting metabolic rate (RMR) than women after a bout of endurance exercise [37]. It would seem that women are better able to resume normal resting metabolic parameters after exercise, whereas, metabolism of men remains changed to a significant degree for longer. It is possible that men undergo a higher degree of respiratory uncoupling or a higher metabolic burden when processes such as lipolysis and gluconeogenesis take place after exercise. This could be viewed as there being a higher level of precise metabolic control in women than in men, as women reach the resting rates of lipolysis and hepatic glucose production after exercise much quicker than men [33,37,38].

In our studies using 25–26 months old mice that had undertaken regular treadmill exercise during middle-to-old age, a significant gender difference in resting metabolism after exercise was observed (Figure 1B). As noted above, exercised male mice tend to use carbohydrates as a fuel source during the dark (active) hours. By way of contrast, in female mice, RER was relatively unaffected during the active hours; however, exercised female mice do tend to consume more fat as an energy source (RER < 0.85, Figure 1B) during the light (sleep) hours. These findings clearly show that there is a greater reliance among female mice than among male mice on fat as an energy substrate during rest post exercise [33].

## 4. Pro-Longevity Genes

### Long-Lived Mouse Models

A long-lived phenotype was first observed in the growth-retarded Ames dwarf mice and Snell dwarf mice, which have mutations of the *Prop1* and *Prou1f1* (Pit1) genes, respectively [39,40,41]. Later, these observations were able to link lifespan control and the best studied aging signaling pathway, the GH (growth hormone)/IGF-1(insulin-like growth factor-1)/Insulin cascade. Furthermore, extended lifespan has been observed in genetic engineered mouse models where there was loss of genes involved in insulin signaling, examples being GHR/BP (growth hormone receptor/GH binding protein) knockout (KO) mice [42], Igf1r (insulin-like growth factor type 1 receptor) heterozygous KO mice [43,44,45], liver-specific Igf1 KO mice [46], Irs1 (Insulin receptor substrate 1) KO mice [47], neuron-specific Irs2 (Insulin receptor substrate 2) KO mice [48] and adipocyte-specific insulin receptor KO mice [49]. Moreover, an indirect suppression of Irs1 and Irs2 via inactivation of S6K1 (ribosomal protein S6 kinase 1) in mice was found to lead to an increased lifespan [50]. Another long-lived mouse model is the Sirt6 transgenic mouse model, where lower serum levels of Igf1 are found, and there are alterations in the phosphorylation levels of major components of the IGF1 signaling pathway [51]. In addition, upstream hormone-like factors, such as Fgf21 (fibroblast growth factor 21) overexpression [52], hNAG-1/Gdf15 (growth differentiation factor 15) overexpression [53] and MIF (macrophage migration inhibitory factor) loss of function [54], have been found to extend the lifespan of mice by reducing the serum level of Igf1. Despite of these observations, it remains under debate whether insulin resistance or insulin sensitivity is better linked to longevity [43,50,52,53,55,56,57].

In addition to the GH/IGF/insulin pathway, a growing number of long-lived mouse models have provided further clues to the metabolic alterations that occur during aging, though the molecular mechanisms of these networks are tightly connected. Overexpression of a thermogenic gene Ucp1 (uncoupling protein 1) in muscle is able to reduce body weight and body fat mass; this is brought about by creating an increase in body temperature. The ectopic overexpression of Ucp1 mimics nutritional deprivation by bringing about activation of AMPK, as well as inactivation of the mTOR signaling cascade; these lead to an increase of medial lifespan of 3 months [58]. Another report showed that a deficiency in cAMP-dependent protein kinase A (PKA) in mice leads to an extended lifespan, increased metabolic activity and a higher body temperature [59]. Pten transgenic mice are also long-lived, and show an elevated resting metabolic rate, as well as reduced fat mass. The overexpression of Pten activates the expression of Ucp-1 and Pgc-1a, which result in a concomitant increase in glucose uptake and oxygen consumption by brown adipose tissue [60]. Another example of a long-lived mouse occurs when there is brain-specific overexpression of Sirt1 (i.e., BRASTO). Aged BRASTO mice display significantly enhanced physical activity, a higher body temperature and increased oxygen consumption; these effects have been attributed to Sirt1-mediated orexin type 2 receptor (Ox2r) upregulation and the colocalization of Ox2r with Sirt1 in the dorsomedial and lateral hypothalamic nuclei [61]. In the genetically modified mice described above, one common feature is a decreased body mass that is accompanied by reduced adiposity [46,49,50,53,58,59,62]. The casual relationship between metabolism and lifespan is still not fully understood and this is despite the observation that mice with a high metabolic activity show greater mitochondrial uncoupling at a young age and live longer [63]. In addition to genetic modification, an evolutionarily-conserved and highly reproducible intervention that is able to extend lifespan is calorie restriction. In mice, diet restriction at either 1-month-old or 1-year-old changes the systemic metabolism of the mice, leading to a prolonged lifespan [54,64]. Exercise also alters metabolism in an organism [65,66]. Pharmaceutical research has shown that a mitochondrial uncoupler, namely 2,4-dinitrophenol, decreases the efficiency of energy conversion in a similar manner to caloric restriction and this can promote in various tissues enhanced tissue respiratory rates, improved serological glucose levels, better triglyceride levels, better insulin levels, decreased reactive oxygen species levels, reduced DNA and reduced protein oxidation, as well as reducing body weight. Importantly, 2,4-dinitrophenol-treated animals also presented with enhanced longevity [67].

Removal of harmful intracellular material has also been found to be as a key factor when trying to improve cellular and organismal health, thereby preventing aging. The p66^Shc^ adaptor protein is a newly recognized mitochondrial protein that oxidizes cytochrome c and forms reactive oxygen species (ROS) by utilizing electrons during respiratory chain dysfunction [68]. Mice that have a lower level of p66^shc^ are long-lived, and display increased resistance when subjected to various cellular stresses, including hydrogen peroxide, UV irradiation and paraquat [69]. Mitochondrial catalases are well studied enzymes that play various critical roles in protecting cells against the toxic effect of hydrogen peroxide. A transgenic mouse overexpressing a human mitochondrially-targeted catalase (MCAT) was generated to test the free radical theory of aging. Indeed, the MCAT transgenic mice showed increased resistance against ROS stress and there was also less accumulation of oxidative damage, as well as extended lifespan [70]. Adenylyl cyclase type 5 (AC5) is expressed in striatal medium spiny neurons, and is known to mediate G protein-coupled receptor signaling through the production of the second messenger cAMP [71]. AC5 KO mice are long-lived, are resistant to cardiac stress and have an increased median lifespan of approximately 30% [72]. AC5 KO mice are protected from reduced bone density, are less susceptible to fractures caused by aging and show less age-dependent decline in cardiac function [72]. Activation of autophagy is an alternative signaling pathway for extending lifespan in mice. The autophagy related 5 (ATG5) transgenic mice that have enhanced autophagy show an extension of their median lifespans by 17.2% and are metabolically healthier than the control cohort mice at 18-month old [62]. By expressing the Beclin1 F121A mutation, the interaction between Beclin1 and Bcl2 is disrupted and autophagy is stimulated. This activation of autophagy results in improved age-associated pathology within the kidneys and heart. The medial survival of both male and female Beclin1 F121A mutant mice is extended by 3 months, compared to gender-matched controls [73].

Taking all of the above genetic, behavioral and chemical intervention studies that use mouse models together, their findings indicate that insulin signaling, energy metabolism, the removal of harmful oxidative stress and a reduction in damaged organelles are the keys to longevity.

## 5. Cisd2 in Aging

Cisd2 (CDGSH Iron Sulfur Domain 2) is an oxidative stress-sensitive gene, the expression of which is able to prolong the lifespan in mice [74]. Cisd2 loss-of-function mice exhibit premature aging phenotypes and have a shortened lifespan. Conversely, Cisd2 transgenic mice not only are longer lived (both males and females), they also have a healthier physical condition, such as better fur function, increased muscle strength and improved cardiac function [75,76]. The Cisd2 protein has been localized to mitochondria, mitochondria-associated membrane (MAM) and the endoplasmic reticulum (ER), and is involved in calcium homeostasis [77]. We have demonstrated using aged mice that maintenance of the expression level of Cisd2 sustains metabolic activity, ameliorates aging-associated mitochondrial dysregulation, reduces DNA damages and improves the calcium imbalance within skeletal muscles [75], liver [77] and heart [76]. Taken together, Cisd2 is a lifespan regulator and its expression level seems to be a critical factor in relation to prolong healthspan.

## 6. Cisd2 and Exercise

**Figure 2:**
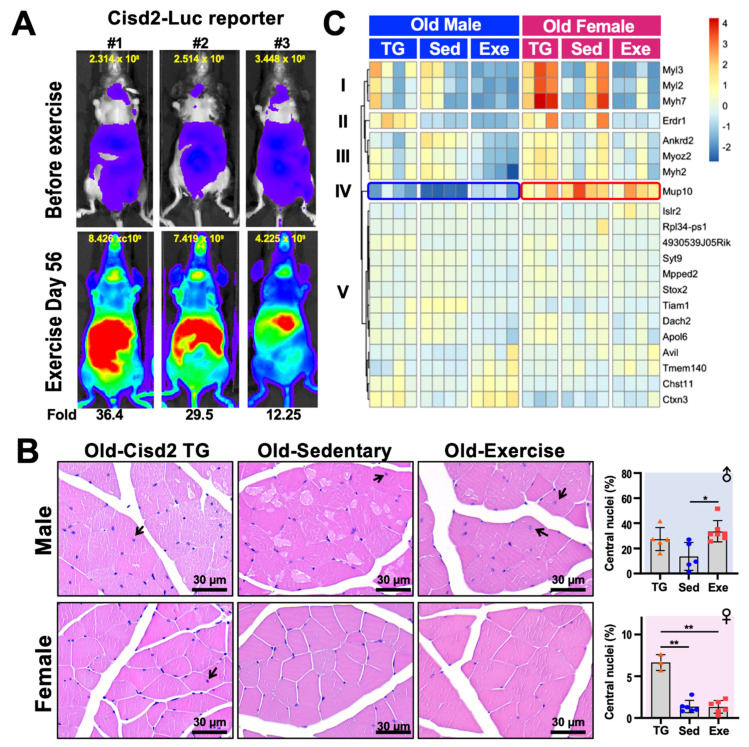
Histology and transcription analysis of femoris muscle from mice that have practiced long-term exercise. (**A**) Luciferase activity in Cisd2-Luc reporter mice before and after 56-days of treadmill exercise. The treadmill protocol was 6 m/min for 5 min, 9 m/min for 5 min, 12 m/min for 20 min, 15 m/day for 5 min and 12 m/day for 5 min, for a total of 450 m/day. The Cisd2-Luc reporter mice ran on treadmills at a frequency of 5 days/week for 8 weeks (56 days). The quantification of luciferase intensity in each of the mice in the figure is shown at top of each image. Increased (fold change) Cisd2-Luc luciferase signals are numbered below the images; these represent mice after the exercise was completed. The photos were taken ventrally. (**B**) H&E staining of the femoris muscles of Cisd2-TG, Old-Sed and Old-Exe male and female mice. The arrows indicate myofibers that contain central nuclei. Bars: 30 μm. Percentage of central nucleation in each group of mice is presented on the right. The data are presented as means ± SD. Statistical analysis used the Student’s t-test. *, *p* < 0.05; **, *p* < 0.01. TG, Cisd2-TG; Sed, sedentary; Exe, exercise. (**C**) A heatmap showed the differentially expressed gene (RNA-Seq) clusters for the male and female femoris muscle that have been changed by long-term treadmill exercise. The RNA-seq data was analyzed by “DEseq2” and the DE genes were defined as LFC > 0.14 and FDR < 0.1. The heatmap was generated using the “pheatmap” R package (Raivo Kolde (2019). pheatmap: Pretty Heatmaps. R package version 1.0.12. https://CRAN.R-project.org/package=pheatmap) according to rlog transformed count calculated by “DEseq2”.

Cisd2 and exercise have both been reported to contribute to an extended lifespan and to improve healthspan. A recent report has shown that the protein levels of Cisd1 and Cisd2 in skeletal muscle and white adipose tissue are increased approximately 1.5-fold and 1.2-fold after 4 weeks of voluntary excise in mice [78]. The authors also observed that there were significant increases in the levels of multiple mitochondrial proteins, which agrees with our previous discovery of increased mitochondrial number in the muscle of Cisd2 transgenic mice compared to their wild type cohorts [75]. To examine the transcription of Cisd2 in real-time, here we have generated a Cisd2 reporter transgenic mouse that carries luciferase as the reporter, which is under the control of the mouse Cisd2 endogenous upstream 31.3-kb promoter sequence (i.e., a Cisd2-Luc mouse). In addition to the endogenous promoter, a 46.9-kb region downstream of Cisd2 coding region was also included in the transgene construct in order to preserve the regulatory elements of the Cisd2 gene. The Cisd2-Luc reporter mice were trained on a treadmill for 56 days. It was found that a drastic enhancement in Cisd2 transcription was able to be observed. The most intense signal was observed at the abdomen, with the thymus showing the next largest increase in signal. A moderately increase in signal was also observed in forelimbs and hindlimbs (Figure 2A).

We further analyzed the histology of the femoris muscle of male and female Old-Sed and Old-Exe mice as described in Figure 1, and compare these to the Cisd2-transgenic (Cisd2-TG) mice at 24–26 months old as reported in Shen et al. [77]. Quadriceps femoris is composed of four individual skeletal muscles, rectus femoris, vastus medialis, vastus lateralis and vastus intermedius. The quadriceps femoris was chosen for this study because of its mass, the fiber type composition and our recent use in other studies. The quadriceps femoris is the largest muscle present in the mouse hindlimb by mass [79]. Furthermore, the quadriceps femoris muscle in terms of fiber types represent the majority of skeletal muscle. The fiber type of the quadriceps femoris muscle is IIx/IIb in rodents and IA/IIA and IIA/IIX in humans, which are commonly referred to as fast-twitch fibers. Most other skeletal muscle in limbs, other than the soleus muscle, have a similar fiber type composition both in humans and other mammals [80]. Finally, the quadricep femoris has often been used to study exercise [80] and the effects of aging [77,81,82] in mouse and humans. In agreement with the results on systemic metabolism (Figure 1B)**,** we found a gender difference in the histology of skeletal muscle, particularly in the Old-Sed mice. In the male mice, multiple vacuolated fibers were observed in the skeletal muscles of these old sedentary mice. This pathology was obviously attenuated in old Cisd2-TG male mice [75] and the Old-Exe male mice (Figure 2B). Additionally, central nucleation was seen in the femoris muscle of Cisd2-TG (27.3 ± 9.15 % of total fiber, *p* = 0.07) and Old-Exe (33.6 ± 8.50 % of total fiber, *p* = 0.01) male mice (Figure 2B, arrows). Central nucleation in muscle cells is an indicator of degeneration followed by regeneration. Accordingly, these findings suggest that exercise and an elevated level of Cisd2 are both able to enhance the repair of muscle fibers. However, in female mice, the age-related pathohistological alterations to the skeletal muscles is less obvious in the Old-Sed group and thus there appears to be no differences in pathology across the three groups of female mice (Figure 2B). Notably, quantification of central nucleation in the female mice reveals that Cisd2-TG mice exhibited a significantly higher ratio of central nuclei in their muscle fibers (6.63 ± 1.0 % of total fiber) (Figure 2B). This suggests that Cisd2 indeed has a beneficial effect on muscle regeneration within both males and females during aging.

To better understand the alternations in the transcriptome that are brought about by long-term exercise and/or Cisd2 overexpression, as well as which signaling pathways are potentially involved during muscle aging, we performed RNA-seq analyses by DEseq2 [83] on femoris muscles obtained from the various groups of mice, namely Old-Exe, Old-Sed and Cisd2-TG (24–26 months old) mice. Due to the substantial individual variation often observed in tissue samples collected from aged mice, a less stringent criteria [log2 fold change (LFC) > 0.14 and false discovery rate (FRD) < 0.1] were set in order to identify the differentially expressed (DE) genes. Comparing Old-Exe and Old-Sed femoris muscle, 19 DE genes and two DE genes were identified in males and females, respectively. On the other hand, only one DE gene, namely *4930539J05Rik*, was found when Cisd2-TG and Old-Sed mice were compared among males, and no DE gene was identified among females. These findings correlate with the macroscopic observations obtained by histopathological analysis (Figure 2B)**.**

A heatmap of the DE genes was plotted to explore the expression pattern of these genes across the three groups (i.e., Cisd2-TG, Old-Sed and Old-Exe, Figure 2C). There were 21 DE genes in total and these were able to be grouped into five clusters. Cluster I, II and III consisted of the DE genes that are significantly down-regulated in the Old-Exe group, compared to the Old-Sed group, and most of those DE genes encode sarcomere proteins. Of these, *Myl3* (LFC = −5.48, FDR = 0.01), *Myl2* (LFC = −4.3, FDR = 0.06) and *Myh7* (LFC = −4.94, FDR = 0.09), encode the slow-twitch type myosin, and *Myh2* (LFC = −2.61, FDR = 0.001), encodes fast-twitch type of myosin [84,85,86]; *Myoz2* is a gene mediating fiber type switching, and *Myoz2* deficiency has been reported to cause an increase in slow-twitch muscle fibers [87]; *Ankrd2* encodes a I-band structure protein that also participates in embryonic myoblast differentiation [88,89,90].

The expression profile of *Mup10*, the only gene in cluster IV, is particularly interesting. Compared to male Old-Sed mice, *Mup10* is significantly up-regulated in Old-Exe (LFC = 3.54, FDR = 0.008) and Cisd2-TG (LFC = 2.98, FDR = 0.201) male femoris muscle (Figure 2C, blue frame). Mups (major urinary proteins) form a highly polymorphic gene family that are expressed in the liver. They are secreted into the bloodstream in order to regulate pheromone stability, and urine Mup profiles are able to function as individual identity signatures of the owners. Circulating MUPs serve as a metabolic signal that links the regulation of glucose and lipid metabolism [91]. Whereas the protein function of Mup10 has not been extensively explored, recombinant MUP1 has been shown to elevate energy expenditure markedly, ameliorate hyperglycemia and reduce glucose intolerance in mice with type 2 diabetes [91]. Intriguingly, the level of *Mup10* transcripts in females is higher overall than in males (Figure 2C, red frame), whereas many other reported MUPs show much higher transcription levels in males than in females [91]. These findings suggest that gender dimorphism of Mup10 in rodents is significantly associated with exercise.

Differences of the 13 DE genes in cluster V were moderate between the three experimental groups. Among these genes, *Islr2* (LFC = 2.05, FDR = 0.005) and *Rpl34-ps1* (LFC = −22.09, FDR < 0.001) are the DE genes in the female Old-Exe group compared to the gender-matched Old-Sed group, while 4930539J05Rik (LFC = 2.39, FDR < 0.001) is the only gene differentially expressed in male Cisd2 TG muscle compared to Old-Sed muscle. In addition, *Rpl34-ps1* can be seen to have a gender difference in its expression profile across exercised mice; this gene is significantly up-regulated in male Old-Exe (LFC = 15.8, FDR < 0.001) but down-regulated in female Old-Exe (LFC = −22.09, FDR < 0.001). Finally, *Avil* (LFC = 1.48, FDR = 0.06), *Tmem40* (LFC = 1.34, FDR = 0.07), *Chst11* (LFC = 1.08, FDR = 0.07) and *Ctxn3* (LFC = 1.66, FDR < 0.001) are all up-regulated in Old-Exe femoris muscle samples compared to the Old-Sed group. Of the last group of genes, it is known that *Chst11* (carbohydrate sulfotransferase), a Wnt/β-cateinin down-stream target [92], participates in chondrocyte development and cartilage morphogenesis [93]. Collectively, our findings reveal that at middle-to-old age treadmill exercise creates a clear gender dimorphism in the transcriptome of the femoris muscle, and that this specifically bring about and causes expression changes in fast-twitch myosins, slow-twitch myosins and the secreted/excreted protein Mup10.

## 7. Conclusions and Perspectives

**Figure 3:**
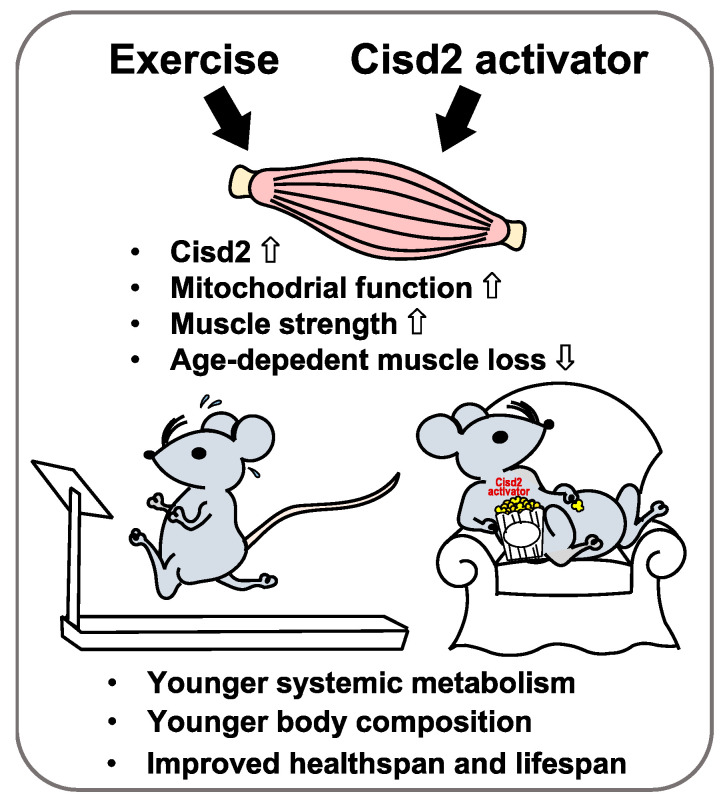
Beneficial effects of exercise and Cisd2 overexpression delay aging. Exercise induces expression of Cisd2, as well as other mitochondrial proteins, in skeletal muscle. Both exercise and Cisd2 overexpression lead to improved mitochondrial function, greater muscle strength and reduced aging-associated muscle loss. They further bring about a reversal of the systemic metabolism and body composition changes associated with aging and return the mice to a status closer to that of young individuals. Taking the evidence together, it seems that exercise and Cisd2 activation are two very promising strategies as an individual ages to build a healthy lifespan.

It has been well demonstrated in multicellular organisms that damage and other deleterious changes accumulate in evolutionarily conserved manner over time and these lead to aging. These changes are often irreversible; however, using genetically-modified mouse models, it has been discovered that a number of major molecular pathways, including insulin signaling, energy metabolism and removal of harmful oxidative stress, seem to govern longevity. Regular exercise has been well demonstrated to improve insulin sensitivity, increase metabolic function and induce autophagy across multiple organs. Here we have further demonstrated that metabolic function is improved in very old male and female mice that undertake treadmill exercise at middle-to-old age, although significant gender dimorphism is also obvious. These exciting discoveries, which relate exercise, metabolic regulation and healthspan, will help us to find new avenues that can be used as actionable interventions against aging (Figure 3).

## Abbreviations

AC5	Adenylyl cyclase type 5
Ankrd2	ankyrin repeat domain 2 (stretch responsive muscle)
ATG5	Autophagy related 5
Avil	Advillin
Bcl2	B cell leukemia/lymphoma 2
Chst11	Carbohydrate sulfotransferase 11
Cisd1	CDGSH iron sulfur domain 1
Cisd2	CDGSH iron sulfur domain 2
Ctxn3	Cortexin 3
DE	Differentially expressed
DEXA	Dual energy X-ray absorptiometry
EE	Energy expenditure
ER	Endoplasmic reticulum
FFA	Free fatty acid
Fgf21	Fibroblast growth factor 21
FRD	False discovery rate
GH	Growth hormone
GHR/BP	Growth hormone receptor
Gdf15	Growth differentiation factor 15
HGPS	Hutchinson-Gilford progeria syndrome
IGF-1	Insulin-like growth factor-1
Irs1	Insulin receptor substrate 1
Irs2	Insulin receptor substrate 2
Islr2	immunoglobulin superfamily containing leucine-rich repeat 2
LFC	Log2 fold change
MAM	Mitochondria-associated membrane
MCAT	Mitochondrially-targeted catalase
MIF	Macrophage migration inhibitory factor
MRI	Magnetic resonance imaging
Mup10	Major urinary protein 10
Myh7	Myosin, heavy polypeptide 7, cardiac muscle, beta
Myl2	Myosin, light polypeptide 2, regulatory, cardiac, slow
Myl3	Myosin, light polypeptide 3
Myoz2	Myozenin 2
Ox2r	Orexin type 2 receptor
Pgc-1a	Peroxisome proliferator-activated receptor gamma coactivator-1 alpha
Pou1f1	POU domain, class 1, transcription factor 1
PKA	Protein kinase A
Prop1	Paired like homeodomain factor 1
Pten	Phosphatase and tensin homolog
REE	Resting energy expenditure
RER	Respiratory exchange ratio
ROS	Reactive oxygen species
Rpl34-ps1	Ribosomal protein L34, pseudogene 1
S6K1	Ribosomal protein S6 kinase 1
TEE	Total energy expenditure
Tmem40	Transmembrane protein 40
Ucp1	Uncoupling protein 1
